# NEDD9 Is a Positive Regulator of Epithelial-Mesenchymal Transition and Promotes Invasion in Aggressive Breast Cancer

**DOI:** 10.1371/journal.pone.0022666

**Published:** 2011-07-28

**Authors:** Chenfei Kong, Changqing Wang, Liping Wang, Musong Ma, Chunbo Niu, Xiaoqian Sun, Juan Du, Zhixiong Dong, Shan Zhu, Jun Lu, Baiqu Huang

**Affiliations:** 1 The Institute of Genetics and Cytology, Northeast Normal University, Changchun, People's Republic of China; 2 The Breast Surgery, The Tumor Hospital of Jilin Province, Changchun, People's Republic of China; 3 The Pathology Department, The Bethune Hospital of Jilin University, Changchun, People's Republic of China; 4 The Key Laboratory of Molecular Epigenetics of Ministry of Education, Northeast Normal University, Changchun, People's Republic of China; Sanford-Burnham Medical Research Institute, United States of America

## Abstract

Epithelial to mesenchymal transition (EMT) plays an important role in many biological processes. The latest studies revealed that aggressive breast cancer, especially the triple-negative breast cancer (TNBC) subtype was frequently associated with apparent EMT, but the mechanisms are still unclear. NEDD9/HEF1/Cas-L is a member of the Cas protein family and was identified as a metastasis marker in multiple cancer types. In this study, we wished to discern the role of NEDD9 in breast cancer progression and to investigate the molecular mechanism by which NEDD9 regulates EMT and promotes invasion in triple-negative breast cancer. We showed that expression of NEDD9 was frequently upregulated in TNBC cell lines, and in aggressive breast tumors, especially in TNBC subtype. Knockdown of endogenous NEDD9 reduced the migration, invasion and proliferation of TNBC cells. Moreover, ectopic overexpression of NEDD9 in mammary epithelial cells led to a string of events including the trigger of EMT, activation of ERK signaling, increase of several EMT-inducing transcription factors and promotion of their interactions with the E-cadherin promoter. Data presented in this report contribute to the understanding of the mechanisms by which NEDD9 promotes EMT, and provide useful clues to the evaluation of the potential of NEDD9 as a responsive molecular target for TNBC chemotherapy.

## Introduction

Breast cancer is a heterogeneous disease, classifiable into five major biologically subtypes, i.e., luminal A, luminal B, basal, ERBB2-overexpressing and normal-like [1], [2], [3]. Importantly, this molecular taxonomy has significant clinical value because some of the molecular phenotypes (especially Her2 and basal-like) show aggressiveness and unfavorable prognosis [2], [3]. The triple-negative breast cancer (TNBC), which accounts for 15%–20% of total breast cancer patients, shares many similarities with the basal subgroup [4], [5]. It refers to any breast cancers that do not express the genes for estrogen receptor (ER), progesterone receptor (PR) and Her2/neu. The bulk of data indicate that this subgroup of patients may have a poorer prognosis than those who with hormone receptor-positive or Her2/neu-positive genotypes [6], [7]. Therapy of TNBC has been a challenge to the physicians because it is resistant to many effective therapeutic approaches. So far, much of the research interest has been focused on identification of new biomarkers of TNBC, but the understanding of its molecular events is still limited. Results from only a few studies suggested that FOXC2, ID1 and LSD1 were involved in the metastasis of TNBC [8], [9], [10]. Weinberg and colleagues found that expression of FOXC2 was induced in cells undergoing epithelial-mesenchymal transition (EMT), and FOXC2 was correlated with the highly aggressive basal-like subtype of human breast cancers [10]. Moreover, LBX1, an EMT inductor, was shown to be upregulated in the triple-negative basal-like subtype [11]. These studies implicated that EMT played a critical role in the invasion and metastasis of TNBC.

A number of studies suggest that carcinoma cells often activate a trans-differentiation program termed the epithelial-mesenchymal transition (EMT) to acquire the ability to execute the multiple steps of the invasion-metastasis cascade [12], [13]. During an EMT, epithelial cells lose cell-cell contacts and cell polarity, express the mesenchymal markers, and undergo major changes in the cytoskeleton that enables cells to acquire a mesenchymal appearance with increased motility and invasiveness [14], [15], [16]. EMT process can be induced by several crucial signaling pathways including the TGF-β [17], Wnt [18] and Notch [19]. Certain developmental factors, such as Snail, Slug, ZEB1 and FOXC2, were also demonstrated to regulate EMT [20].

In recent years, NEDD9 has been confirmed to contribute to the development of several cancer types [21], [22]. Recent studies showed that Nedd9-null genetic background significantly limited mammary tumor initiation in the MMTV-polyoma virus middle T genetic model, suggesting that NEDD9 expression played an important role in breast cancer [23]. Despite these diverse reports, the precise functions and the mechanistic action of NEDD9 have not been well defined. In this study, we demonstrate that NEDD9 is a potent activator of EMT. In mammary epithelial cells, NEDD9 can activate ERK signaling, increase the expression of EMT-inducing transcription factors Snail/Slug and their interactions *in vivo* with the E-cadherin promoter. Ectopic overexpression of NEDD9 led to morphological transformation, induced characteristic molecular features of EMT and enhanced cellular migration, invasion and proliferation. Analysis of NEDD9 expression across different cancers revealed an apparent correlation of this gene with the aggressive human triple-negative breast cancer.

## Materials and Methods

### Ethics statement

Written informed consent was obtained from all participants involved. We obtained ethics approval from the ethics committees at The Tumor Hospital of Jilin Province and The Bethune Hospital of Jilin University.

### Tissue specimens and cell culture

Breast carcinoma tissues were obtained from the Tumor Hospital of Jilin Province and the Bethune Hospital of Jilin University. Samples were frozen in liquid nitrogen immediately after surgical removal and maintained at −80°C until use. All human tissues were collected using the protocols approved by the Ethics Committee of the Jilin Tumor Hospital. The normal human breast epithelia cell lines and the human breast cancer cell lines were obtained from the Institute of Cell Biology, Shanghai, China.

### Plasmid constructs and transfection

The E-cadherin promoter plasmid was a gift from Dr. Ji-Hshiung Chen (Graduate Institute of Molecular and Cell Biology, Tzu Chi University, Taiwan). The LZRS-Ires-Nedd9 plasmid was generously provided by Dr. Lynda Chin (Department of Dermatology, Harvard Medical School, Boston). Nedd9 cDNA was cloned using the following primers: 5′-CCGCTCGAGATGTGGACAAGGAATCTTATGGC-3′ (sense) and 5′-CCGGAATTCAGAACGTTGCCATCTCCAGCAAAGA-3′ (antisense). The resultant DNA fragment was inserted to pEGFP-N1 vector at *Xho*I and *Eco*RI sites. Short interfering RNA (siRNA) targeting the Nedd9 sequence (GAAGCTCTATCAAGTGCCA) was synthesized. Oligonucleotide that represents the siRNA was cloned into the pSuper-neo vector (Oligoengine) between *Eco*RI and *Hind*III sites following the manufacturer's instructions. NEDD9-GFP and pEGFP-N1 were transfected to the MDCK and MCF10A cells using FuGENE HD (Roche) following the manufacture's instructions. Cells were selected with G418 for more than two weeks to establish NEDD9-MDCK, EGFP-MDCK, NEDD9-MCF10A, EGFP-MCF10A. NEDD9 siRNA and control siRNA were transfected to the MDA-MB-231 cells using Amaxa nucleofector kits (Lonza). Cells were selected with G418 for more than two weeks to establish NEDD9 siRNA-MDA-MB-231, control siRNA-MDA-MB-231.

### Wound-healing assay

Cells (1×10^6^ cells per well) were seeded on 6-well plates. 24 hr later, cell layers were wounded in serum-free medium with 1% bovine serum albumin using a sterile 200 ml pipette tip. After washing away the suspended cells, cells were subjected to serum starvation. The progress of migration was photographed in six regions, immediately and during 2 days after wounding (0/12/24/48 hr), under an inverted microscope.

### RNA extraction, reverse transcription and quantitative RT-PCR

Total RNA was isolated using the Trizol reagent (Invitrogen) following manufacturer's instructions. One microgram RNA was used for cDNA synthesis using a reverse transcriptase reaction kit (Promega). Quantitative real-time RT-PCR was carried out on an ABI Prism 7000 Sequence Detection System (Applied Biosystems), and SYBR Green (TOYOBO) was used as a double-stranded DNA-specific fluorescent dye. The PCR primer sequences were mentioned in the Methods S1.

### Western blotting

Western blotting was performed as described previously [24]. Monoclonal anti-NEDD9 (ab18056), anti-snail (ab63371), anti-slug (ab27568) was purchased from Abcam (Cambridge, USA). Monoclonal anti-vimentin (v6630) and anti-β-actin were purchased from Sigma (St. Louis, Missouri). Monoclonal anti-fibronectin (610077) was purchased from BD Biosciences (California, USA). Monoclonal anti-occludin (33–1500) was purchased from Invitrigen (Invitrigen, USA). Monoclonal anti-p44/42 MAP Kinase (137F5) and phospho-p44/42 MAPK Thr202/Tyr204 (197G2), polyclonal anti-E-cadherin (#4065) and anti-N-cadherin (#4061) antibodies were purchased from CST (USA).

### Immunofluorescence

Cells were grown on glass cover-slips in a six-well plate and washed three times with PBS then fixed in 4% formaldehyde solution and permeabilized with 0.1% Triton X-100 in PBS for 5 min. Cells were blocked with 2% BSA in PBS for 30 min at room temperature. Cover-slips were incubated with respective primary antibodies at 1∶100 dilutions for 1 hr and then washed with PBS and incubated for 1 hr with TRITC-conjugated secondary antibodies at 1∶50 dilutions (Zhongshan, China). Cells were further washed in PBS and mounted with Vectashield mounting medium containing 4′, 6-diamidino-2-phenylindole (DAPI; Vector Laboratories) and were analyzed using fluorescence microscopy. Photographs were taken under a Nikon microscope with a fluorescein isothiocyanate filter.

### Cell migration and invasion assay

*In vitro* cell migration assays were performed as described previously [25]. Images of three random ×10 fields were captured from each membrane and the number of migratory cells was counted. The means of triplicate assays for each experimental condition were used. Similar inserts coated with Matrigel were used to determine invasive potential in the invasion assay.

### MTT assay

Cell proliferation was assessed by using the MTT [3-(4, 5-dimethylthiazol-2-yl)-2, 5-diphenyl tetrazolium bromide] assay. Cells were plated at 1×10^5^ cells/well on 96-well plates. At 24, 48 hr after transfection, 20 µl of MTT (5 mg/ml) was added to each well; the samples were incubated for 4 hr at 37°C and then sub-cultured to the medium with 100 µl dimethyl sulfoxide (DMSO). The absorbance of each well was determined at 492 nm. Survival percentage (%) was calculated relative to the control.

### Colony formation assay

Cells were plated in 10-cm tissue culture plates 24 hr before transfection. pEGFP-N1 control vector or NEDD9-GFP expression vector was transfected. 24 hr later, the transfected cells were diluted, re-plated, and selected in 10-cm plates containing 1 mg/L G418 for 12 days. Colonies were stained with crystal violet (Sigma-Aldrich).

### Luciferase reporter assay

Reporter gene assays were done as previously described [24]. Briefly, 5×10^4^ cells were seeded in 24-well tissue culture plates 24 hr before transfection. The E-cadherin promoter luciferase reporter was transfected at 100 ng/well and the *Renilla* luciferase control plasmid pREP7-RLuc was cotransfected at 50 ng/well as an internal control reporter. For reporter assays in HEK293T cells, β-catenin was used to activate the reporter gene. Increasing amounts of NEDD9-GFP expression vector were transfected into cells. Thirty hours post transfection, cells were washed and lysed in passive lysis buffer (Promega) and the transfection efficiency was normalized to the paired *Renilla* luciferase activity by using the Dual Luciferase Reporter Assay System (Promega) according to the manufacture's instructions.

### Chromatin immunoprecipitation assay

The protocol for chromatin immunoprecipitation (ChIP) was described elsewhere [24]. Briefly, the chromatin solution was precleared with 50 µl of protein A-agarose beads (Upstate Biotechnology). The soluble fraction was collected and 5 µg of antibodies was added. The precipitated chromatins were analyzed by PCR. Primer 1 and 2 were used to amplify the E-cadherin promoter regions from −600 to −329 and −359 to −63, respectively. A human negative control was designed. The primers used were: P1, sense: 5′-TGGTGGTGTGCACCTGTACT-3′, antisense: 5′- GACCTGCACGGTTCTGATTC-3′; P2, sense: 5′- CGAACCCAGTGGAATCAGAA -3′, antisense: 5′- GCGGGCTGGAGTCTGAACTG -3′; and the human negative control sense: 5′-ATGGTCAACCCCACCGTG-3′, antisense: 5′- TGCAATCCAGCTAGGCATG-3′.

### Statistical analysis

Student test was used to calculate the statistical significance of the experimental results. The significance level was set as *P<0.05 and **P<0.01. Error bars denote the standard deviations (SDs).

## Results

### NEDD9 was overexpressed in human aggressive breast cancers

**Figure 1:**
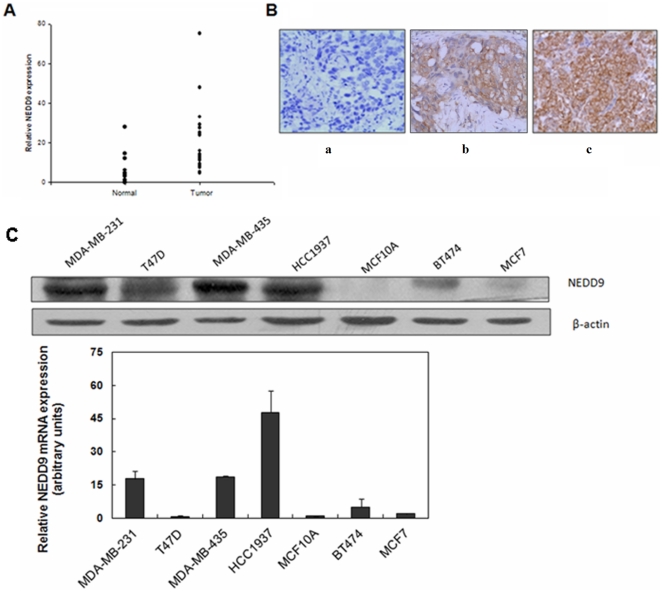
Expression of NEDD9 in breast tissue samples. A, NEDD9 levels in normal breast and breast cancer tissues. Normalized NEDD9 mRNA expression was measured by quantitative RT-PCR with β-actin expression as the internal control. B, Representative IHC of NEDD9 protein expression in paraffin-embedded human primary breast cancer and adjacent normal tissues. *a*, faint cytoplasmic staining of NEDD9; *b*, moderate cytoplasmic staining of NEDD9; and *c*, strong cytoplasmic staining of NEDD9. C, Western blotting and qRT-PCR analysis of endogenous NEDD9 expression in normal breast epithelial cell lines and in breast cancer cell lines. β-actin was used as a loading reference.

NEDD9 was expressed in several types of tumors [21], [22], [26]. This observation prompted us to investigate whether NEDD9 was also overexpressed in breast cancers. We analyzed 20 breast tumor samples, together with their adjacent normal tissues, from breast cancer patients. The results revealed a statistically significant increase in NEDD9 mRNA expression in tumors, compared with their adjacent normal mammary tissues (Fig. 1A). We then employed immunohistochemistry (IHC) to assess the NEDD9 protein expression in the paraffin-embedded mammary tissue sections from 84 breast cancer patients in parallel with the surrounding normal breast epithelia. The results indicated that, while normal mammary epithelial cells displayed none or weak NEDD9 staining (Fig. 1B, a), breast carcinoma cells were positive for NEDD9 staining in cytoplasm and/or in nucleus (Fig. 1B, b and c). Further analysis of the data revealed that NEDD9 expression was associated with several adverse prognostic markers, including estrogen receptor (ER) negativity and high tumor grade (Table S1).

Interestingly, high levels of NEDD9 expression were associated with aggressive breast cancers, including ER^−^/PR^−^/Her2^−^ subtype of invasive ductal breast cancers and Her2/neu-positive breast cancers (Table 1 and Table S1). As shown in Table 1, 31.82% of the TNBC tumors and 24.00% of Her2^+^ subtype tumors exhibited high levels of NEDD9 expression, whereas only 11.62% of the common ER^+^ subtype of tumors showed high expression of NEDD9 protein. To date, only a few distinct molecular markers have been identified that are uniquely associated with TNBC [10], [27], [28], [29]. NEDD9 expression therefore may prove to be a useful diagnostic marker for this subtype. Moreover, western blotting analysis of immunoreactive NEDD9 in established mammary epithelial cell lines indicated that the levels of NEDD9 in aggressive breast cancer cell lines were considerably higher than those in MCF10A cells derived from normal mammary epithelial cells (Fig. 1C). Collectively, these data suggest that NEDD9 is dominantly overexpressed in human aggressive breast cancer.

**Table 1:** The percentage of NEDD9 expression in breast cancers.

	Parameter	High	Low
Non-aggressive	ER^+^(n = 43)	11.62%	88.38%
aggressive	HER2^+^(n = 25)	24.00%	76.00%
	Triple negative(n = 22)	31.82%	68.18%

### NEDD9 was a positive regulator of migration, invasion and proliferation in highly aggressive TNBC cells

**Figure 2:**
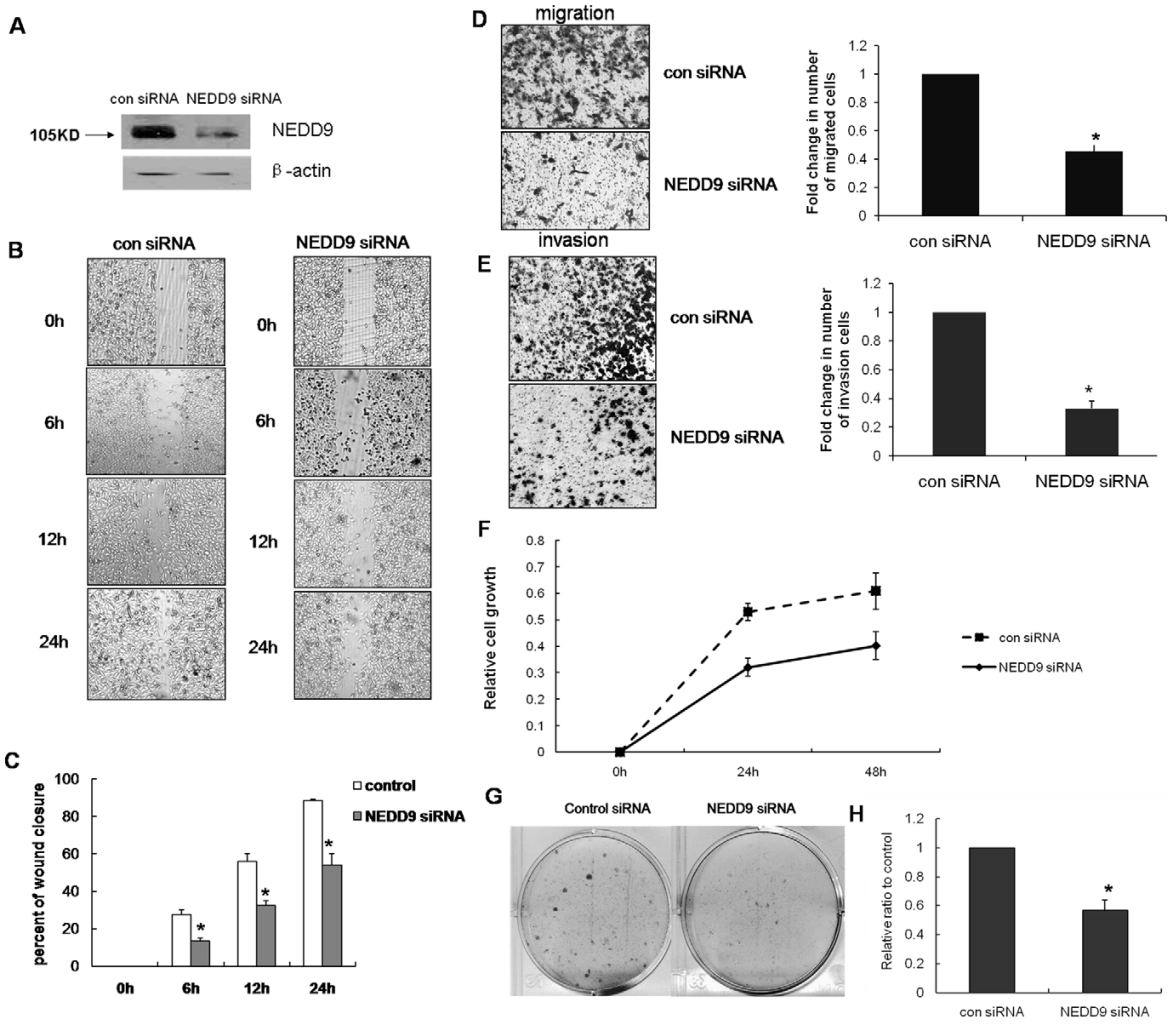
Suppression of NEDD9 expression inhibited tumor cell migration, invasion and proliferation. A, NEDD9 expression in NEDD9 siRNA-MDA-MB-231 cells. The level of NEDD9 in NEDD9 siRNA-MDA-MB-231 and control siRNA-MDA-MB-231 was determined by western blotting. B, C, Knockdown of NEDD9 inhibited MDA-MB-231 cell motility. Cells were plated for a scratch wound assay. Photographs were taken at 0, 6, 12 and 24 hr after wounding. The percentage of wound closure was calculated using Image J software. D, E, Migration and invasion assays using either control siRNA-MDA-MB-231 or NEDD9 siRNA-MDA-MB-231 cells. The migration and invasion ability is presented as fold change in number of cells migrated to the bottom chamber. Bars represent the mean SEM of samples measured in triplicate, and each experiment was repeated at least three times. F, Effect of NEDD9 on cell proliferation. MTT assay was used to estimate the proliferation at different time points. G, H, Effect of NEDD9 on colony formation. Cells were cultured in the presence of 1 mg/L G418 for 2 weeks. Colonies were stained with crystal violet.

**Figure 3:**
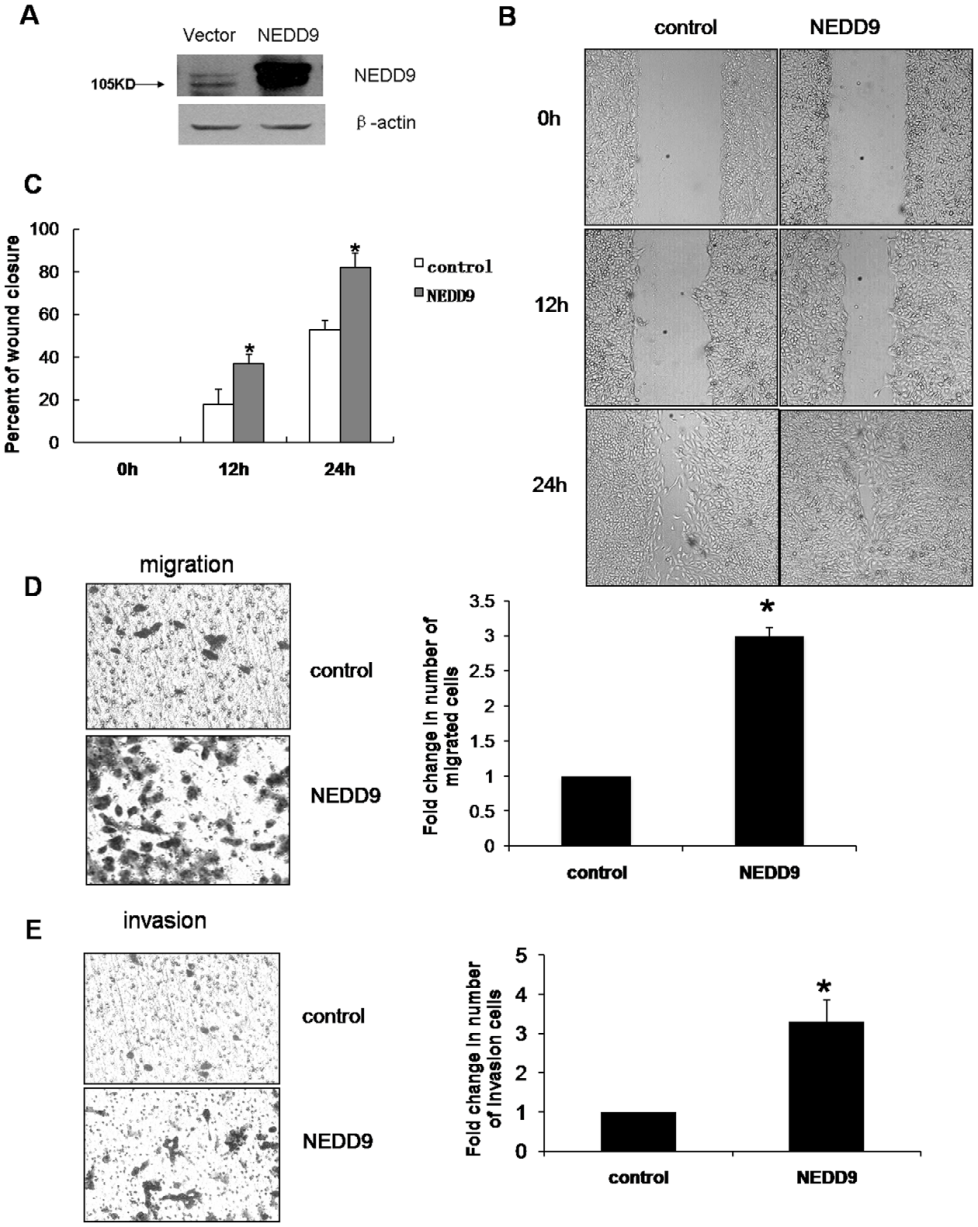
Ectopic expression of NEDD9 enhanced the migration and invasion in MCF10A cells. A, Stable overexpression of NEDD9 in MCF10A cells. The expression level of NEDD9 in NEDD9-MCF10A cells was determined by western blotting. B, C, NEDD9 enhanced the motility of MCF10A cells. Cells were plated for a scratch wound assay. D, E, Migration and invasion assays in NEDD9-MCF10A and NEDD9-GFP cells.

The above results indicated that the endogenous NEDD9 mRNA level was barely detectable in MCF10A cells, but was expressed in several invasive breast cancer cell lines. This suggests that NEDD9 may also play a role in breast cancer migration and invasion. To validate this, we examined the function of NEDD9 in breast cancer by repressing its expression in two highly aggressive TNBC cell lines, MDA-MB-231 [30] and HCC1937 [31]. To test whether constitutive NEDD9 expression in MDA-MB-231 cells contributes to their oncogenicity, we knocked down the endogenous NEDD9 by a specific siRNA. The efficiency of this gene silencing protocol was confirmed by western blotting (Fig. 2A). As shown in Fig. 2B and C, using scratch wound assay, we showed that the NEDD9 siRNA-MDA-MB-231 cells had only completed a half closure at 24 hr compared to the control siRNA-MDA-MB-231 cells. The trans-well migration assays also demonstrated that the control cells migrated approximately 2 times faster than NEDD9 siRNA-MDA-MB-231 cells (Fig. 2D). To further validate the roles of NEDD9 in regulating cell invasion, we performed trans-well Matrigel invasion assay to assess the ability of cells to invade through the Matrigel layer. As shown in Fig. 2E, NEDD9 siRNA-MDA-MB-231 cells invaded much slower than control cells. Moreover, results from MTT assays revealed that stable expression of NEDD9-siRNA inhibited the proliferation of MDA-MB-231 cells (Fig. 2F). Colony formation assays also confirmed that knockdown of NEDD9 expression markedly decreased the number of MDA-MB-231 cell colonies (Fig. 2G, H). In order to rule out the false positive results of migration and invasion caused by proliferation inhibition, we carried out representative assays in the presence of 12 µM mitomycin C (MMC), an alkylating agent which inhibits DNA synthesis. As a result, we found no significant difference between groups treated with and without MMC (Figure. S1). These findings suggested that inhibition of NEDD9 expression reduced the migration, invasion and proliferation of MDA-MB-231 cells. Similar results were obtained with HCC1937 human breast cancer cells when NEDD9 expression was repressed by siRNA (Figure. S2). We next examined the function of NEDD9 in mammary epithelial cells. We showed that overexpression of NEDD9 in MCF10A cell increased cell migration (Fig. 3B–D) and invasion (Fig. 3E).

### NEDD9 promoted epithelial-mesenchymal transition (EMT)

**Figure 4:**
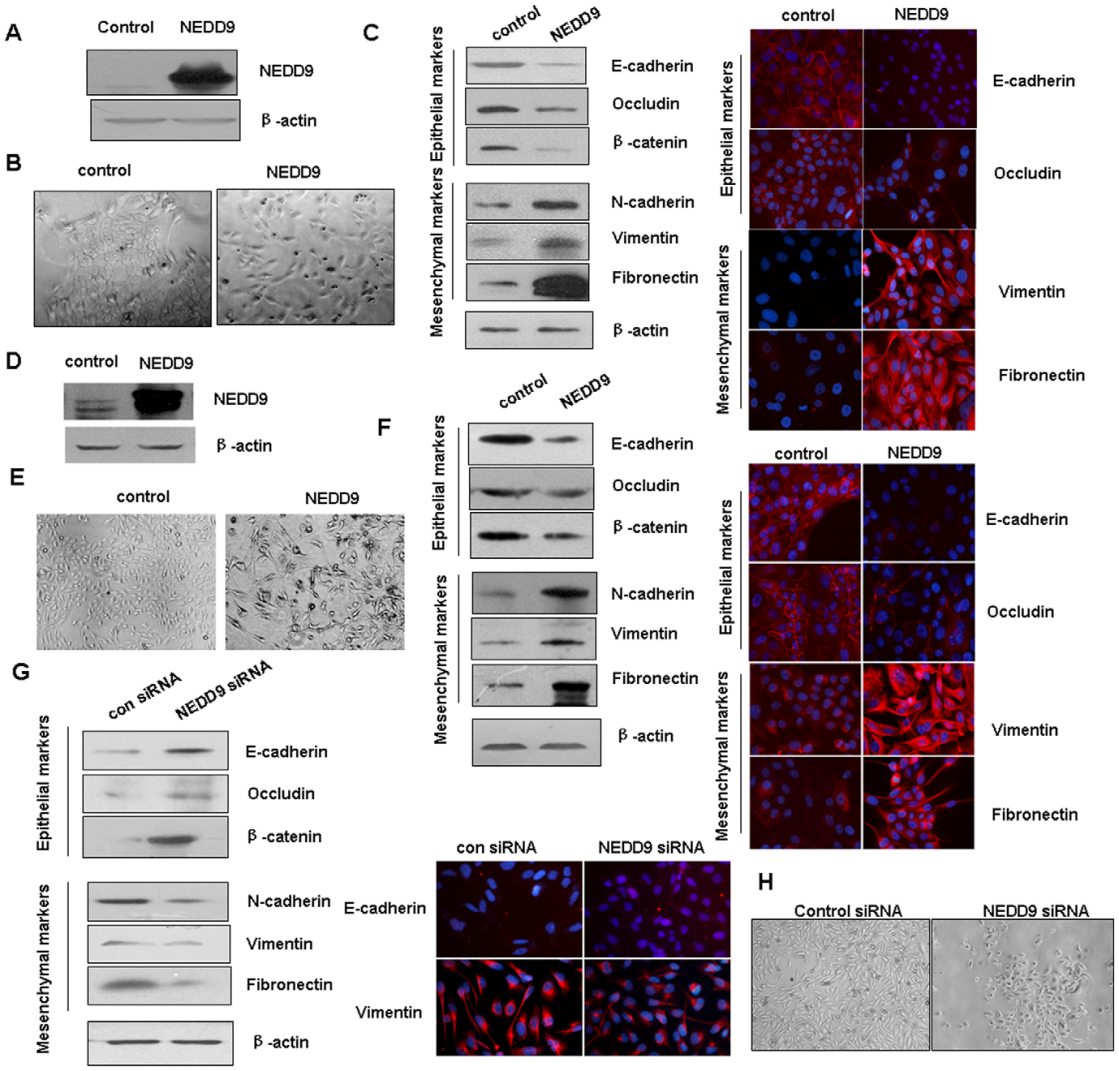
Expression of NEDD9 caused EMT. A, D Stable overexpression of NEDD9 in MDCK and MCF10A cells. The expression level of NEDD9 in NEDD9-MDCK (A) and NEDD9-MCF10A (D) cells was determined by western blotting. B, E, NEDD9-MDCK and NEDD9-MCF10A cells showed the spindle-like, fibroblastic morphology (×20 magnification). C, F, Immunoblots and immunostaining of epithelial and mesenchymal markers in NEDD9-MDCK and NEDD9-MCF10A cells. G, Immunoblots and immunostaining of the epithelial and mesenchymal markers in NEDD9 siRNA-MDA-MB-231 and control cells. H, NEDD9 siRNA-MDA-MB-231 cells displayed an egg-shaped, epithelial-like morphology (×20 magnification).

NEDD9 has been shown to be a target gene of TGF-β cell signaling [32], [33], which is an important signaling in epithelial-mesenchymal transition (EMT), and we found that NEDD9 overexpression in MCF10A mammary epithelial cells changed morphology of the cells (Fig. 4E). So we tested whether NEDD9, on its own, is sufficient to induce the EMT program in a Maden-Darby canine kidney epithelial cell line (MDCK) which has been a widely used model to study epithelial cell biology [34], [35]. First, the NEDD9-MDCK cells were generated and confirmed by immunoblotting (Fig. 4A). As can be seen in Fig. 4B, after ectopic NEDD9 expression, MDCK cells displayed a spindle-like, fibroblastic morphology, one of the main characteristics of EMT. At the molecular level, expression of both epithelial and mesenchymal molecular markers was confirmed by western blotting and immunofluorescence (Fig. 4C). It can be seen that the epithelial markers E-cadherin, Occludin and β-catenin were significantly reduced in NEDD9-MDCK cells. Meanwhile, E-cadherin and Occludin were lost from the cell membranes, as revealed by immunofluorescence. In contrast, the mesenchymal markers, Fibronectin, Vimentin and N-cadherin, which are positively correlated with EMT, were dramatically upregulated (Fig. 4C). We then examined whether NEDD9 could induce EMT process in MCF10A human mammary epithelial cells. The NEDD9-MCF10A cells were generated and confirmed by immunoblotting (Fig. 4D). Similarly, we found that the morphology of the cells changed from epithelial to mesenchymal-like (Fig. 4E); and overexpression of NEDD9 in MCF10A cells caused the reduction of the epithelial markers and increase of the mesenchymal markers (Fig. 4F).

Next, we tested whether suppression of endogenous NEDD9 expression is sufficient to reverse the EMT progression. We showed that after knockdown of NEDD9 in MDA-MB-231 cells, the mesenchymal markers N-cadherin, Vimentin and Fibronectin were reduced, whereas the epithelial markers E-cadherin, Occluding and β-catenin were increased, as revealed by western blotting (Fig. 4G). Similar results were obtained by using immunocytochemistry. Concurrently, the NEDD9 siRNA-MDA-MB-231 cells displayed an egg-shaped, epithelial-like morphology (Fig. 4H), consistent with the increase of epithelial markers and the decrease of mesenchymal molecular markers. These results suggest that suppression of NEDD9 in TNBC cells not only reduced migration and invasion but also partially reversed the EMT process.

**Figure 5:**
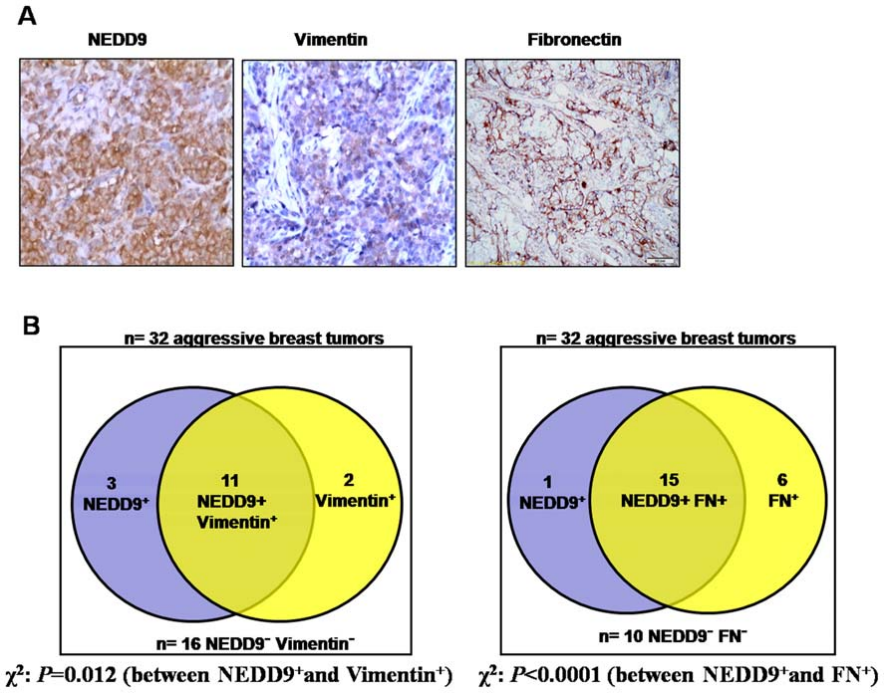
NEDD9 promoted the EMT in aggressive breast tumor. A, Representative immunohistochemical images of an aggressive breast tumor sample stained for NEDD9 and Vimentin. B, Venn diagram showing the association between NEDD9 and Vimentin in aggressive breast tumors.

Finally, we tested whether NEDD9 contributes to EMT *in vivo*. We assessed the correlation between the level of NEDD9 and that of the mesenchymal markers, such as Vimentin and Fibronectin in 32 aggressive breast tumors. We found that positive expression of NEDD9 was significantly associated with the expression of Vimentin and Fibronectin (Fig. 5). Overall, these results demonstrate that NEDD9 is a regulator of EMT.

### NEDD9 promoted EMT through the ERK-Snail/Slug signaling

**Figure 6:**
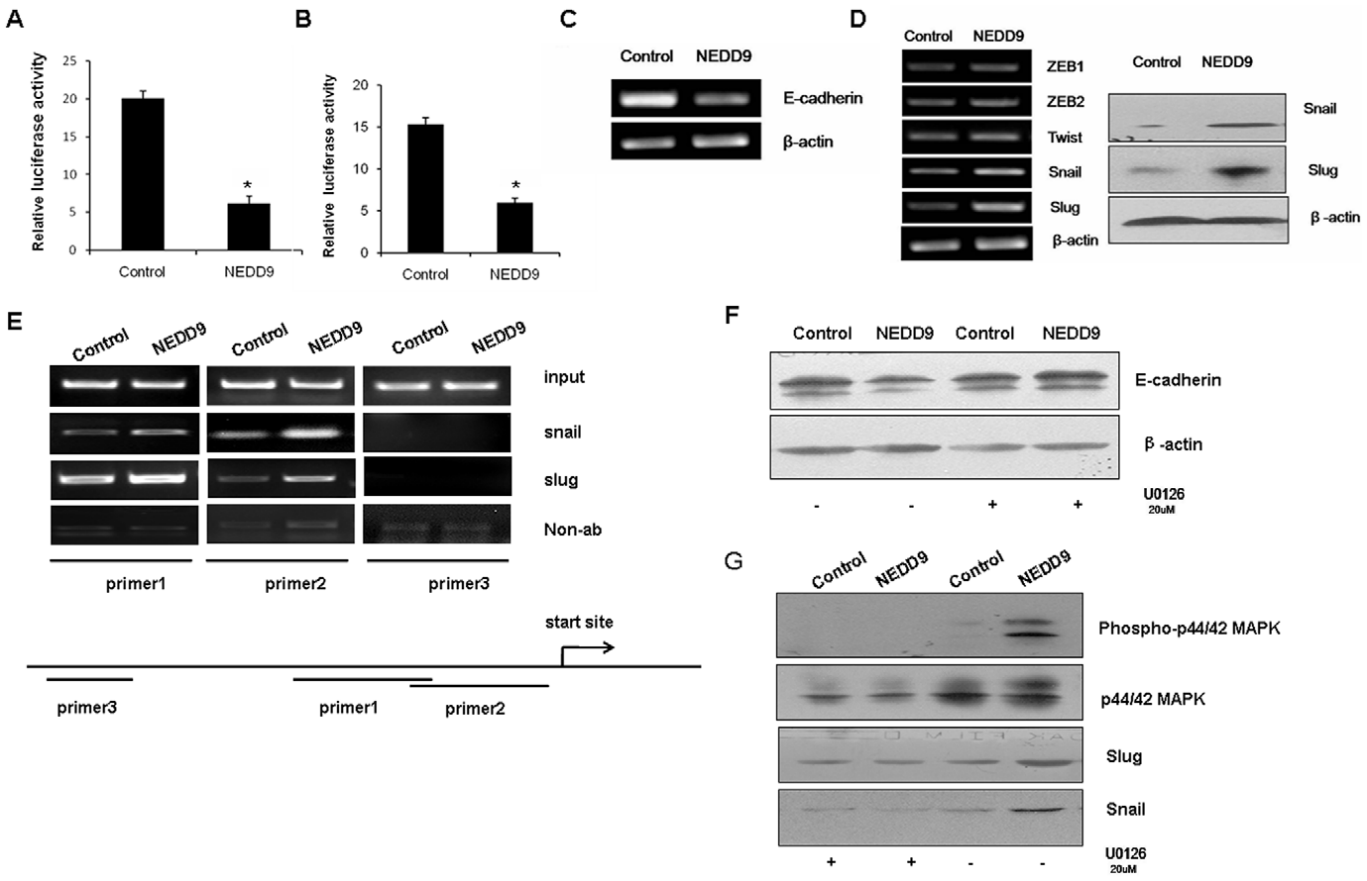
Molecular events involved in NEDD9-induced EMT. A, B, NEDD9 inhibited the E-cadherin promoter activity. HEK 293T cells (*left*) and MCF10A cells (*right*) were transfected with the expression vectors as indicated, and the relative luciferase activity was determined after culturing for 24 hr. C, NEDD9 inhibited the E-cadherin mRNA in MCF10A cells. D, RT-PCR and western analyses of the expression of indicated transcriptional repressors of E-cadherin in NEDD9-MCF10A and control cells. The β-actin was used as an internal control. E, ChIP assays at the E-cadherin promoter. *top*, increased binding of Snail and Slug at the E-cadherin promoter in the presence of NEDD9. *bottom*, map of the E-cadherin promoter showing the sites of the amplification in ChIP analyses. F, G, Western analysis showing the effect of NEDD9 overexpression on ERK activation. Cells were incubated in culture medium containing the indicated concentrations of U0126 (CST) for 24 hr. Lysates were prepared and subjected to western analysis using specific antibodies.

To further investigate the molecular events involved in NEDD9-induced EMT, we first tested whether NEDD9 is capable of interfering with E-cadherin promoter activity by using a human E-cadherin proximal regulatory promoter luciferase reporter gene plasmid. Transient expression of NEDD9 in HEK293T cells resulted in strong downregulation of the activities of reporter gene (Fig. 6A). Similar results were obtained with MCF10A cell line (Fig. 6B). Moreover, mRNA (Fig. 6C) and protein (Fig. 4F) expression levels of E-cadherin were also reduced upon NEDD9 overexpression in MCF10A cells. These data indicate that NEDD9 is a potential factor that downregulates E-cadherin expression during EMT.

Since NEDD9 is not a transcription factor, we wondered whether Snail, Slug, Twist or ZEB, the transcription factors known to repress E-cadherin in various cell systems [36], [37], [38], [39], act in cooperation with NEDD9. RT-PCR analysis in NEDD9-MCF10A and control cells revealed similar expression levels of ZEB1, ZEB2, ID2 and Twist (Fig. 6D). On the other hand, evident increase in Snail and Slug expression was noted in NEDD9 overexpressing MCF10A cells in contrast to control cells, and western analysis confirmed the increased Snail and Slug protein levels (Fig. 6D). Moreover, ChIP assays revealed that overexpression of NEDD9 enhanced the interaction between the Snail family and E-cadherin promoter (Fig. 6E).

It has been reported that activation of the extracellular-signal regulated kinase (ERK) can induce Snail and Slug expression [40], [41]. We then tested if this signaling pathway functions in the cell model we used. We found that treatment of NEDD9-MCF10A cells with 20 µM the specific ERK kinase inhibitor U0126 indeed caused a recovery of E-cadherin expression (Fig. 6F). We further explored the possible involvement of ERK signaling cascade in the NEDD9-mediated regulation of Snail and Slug expression. Western analysis revealed a significant increase of activated ERK (p-ERK) levels in NEDD9-MCF10A cells compared with control cells (Fig. 6G), suggesting that ERK acted downstream of NEDD9.

Together, these data suggest that NEDD9 is able to activate the ERK cascade and subsequently to induce Snail and Slug upregulation that bound to E-cadherin promoter to inhibit its expression. This finally contributes to the EMT and invasive phenotypes of human breast adenocarcinoma cells.

## Discussion

A noticeable point arising from this study is that high levels of NEDD9 expression were associated with the aggressive breast cancers, including TNBC and ERBB2-overexpressing subtypes (Fig. 1, Table 1; Table S1). As shown in Table 1, 31.82% of the triple-negative tumors and 24.00% of Her2^+^ tumors exhibited high levels of NEDD9 expression, whereas only 11.62% of the luminal ER^+^ subtype of tumors showed high expression of NEDD9 protein. High expression of NEDD9 may therefore be a potential diagnostic marker for these subtypes of breast cancer. Our data are compatible with the pro-oncogenic role identified for NEDD9 overexpression in glioblastoma, melanoma, and lung cancers [21], [22], [42]. These studies implicated that NEDD9 may have a promotive effect for the carcinogenic process.

The process of EMT has been shown to provide carcinoma cells with many of the phenotypes required to execute multiple steps of the invasion-metastasis cascade. When examining the NEDD9 and mesenchymal markers, such as Vimentin and Fibronectin in 32 aggressive breast tumors, we found that positive expression of NEDD9 was associated significantly with the expression of Vimentin and Fibronectin (Fig. 5), implicating that NEDD9 may play a role in EMT *in vivo* in aggressive breast tumors. Moreover, we discovered that ectopic expression of NEDD9 promoted migration and invasion in MCF10A cells, and knockdown of NEDD9 in highly aggressive TNBC cells reduced their migration, invasion and proliferation (Fig. 2, 3). These data suggested that NEDD9 was a regulator of the migration, invasion and proliferation in breast cancer cells. In contrast to our findings, a previous study suggested that NEDD9 acted as an inhibitor of migration, as the authors reported that siRNA-mediated NEDD9 depletion promoted cell migration in breast epithelial cells [43]. In line with our results, Fashena et al found that NEDD9 production induced crescent morphology and cell spreading in MCF7 cell lines [44], and Izumchenko et al reported that the Nedd9^−/−^ mice significantly limited the mammary tumor initiation in the MMTV-polyoma virus middle T genetic model [23]. These data support our finding that the NEDD9 is positively correlated with breast cancer progression. However, the authors detected no significant differences when they examined a number of hallmarks of TGF-β-induced EMT in Nedd9^−/−^ tumors and tumor-derived cell lines [23]. These discrepancies may probably be due to the fact that NEDD9 is required at early stages in the EMT process, but downregulated after the metastatic cancers undergo a reverse mesenchymal-epithelial transition (MET) process. Further investigation to address this issue is required.

Our Boyden-chamber trans-well assays determined that the ectopic expression of NEDD9 in MCF10A cells turned this epithelial cell lineage into a highly migratory (Fig. 3D) and invasive (Fig. 3E) phenotypes. Cancer cells degrade the nearby extracellular matrix by using secreted matrix metalloproteinases (MMPs) [45]. As gauged by gelatin zymogram assay and western blotting, we found that MMP9 (Figure. S3), but not MMP2 (data not shown), was upregulated in response to the expression of NEDD9 in MCF10A cells. These observations reinforced the notion that NEDD9 can serve as an organizer of mesenchymal differentiation during an EMT.

Significantly, our data suggest that NEDD9 expression activated the ERK cascade and subsequently induced Snail and Slug upregulation resulted in a potentiated binding of Snail and Slug to E-cadherin promoter (Fig. 6E). These events eventually contributed to the initiation of EMT and invasion in human breast adenocarcinoma cells. Consistent with our results, Storci et al reported that the tumor tissues expressing high levels of Slug mRNA displayed a basal-like breast carcinoma phenotype [46]. Shin et al described that ERK2 specifically regulated EMT in MCF10A cells [47]. ERKs are effectors of MAPK cascade activated by Ras/Raf. Meanwhile, Kim et al demonstrated that NEDD9-dependent tumor promotion was partly dependent on Ras/Raf pathway activation [22]. These studies suggested a close relationship between NEDD9 and Ras signaling in tumor growth.

Moreover, our ChIP assays revealed that overexpression of NEDD9 enhanced the recruitment of histone deacetylase HDAC1/HDAC2 repressor complex to E-cadherin promoter (Figure. S4), indicating that the regulation of E-cadherin repression by overexpression of NEDD9 may involve several epigenetic repressors, although this assumption requires further experiments to confirm.

To summarize, we validated in this study that the expression of NEDD9 was frequently upregulated in highly aggressive TNBC breast cancer cell lines as well as in aggressive breast tumors, including ERBB2-positive and triple-negative subtypes. In vitro, knockdown of NEDD9 reduced the mesenchymal molecular markers, increased the epithelial markers and inhibited the invasion and migration of aggressive TNBC cells. Ectopic overexpression of NEDD9 in MCF10A mammary epithelial cells led to a morphological transformation towards the mesenchymal phenotype, together with the expression of mesenchymal markers, and consequently resulted in an enhanced cell migration, invasion and proliferation. Moreover, ectopic expression of NEDD9 activated ERK signaling, upregulated the expression of the EMT-inducing transcription factors Snail and Slug, and promoted their interactions in vivo with the E-cadherin promoter. Results from this study contribute to the understanding of the mechanisms by which NEDD9 promotes the epithelial-mesenchymal transition. Also, this study provides useful clues to the evaluation of the potentiality of NEDD9 as a responsive molecular target for TNBC therapeutics.

## Supporting Information

Figure S1 **NEDD9 knock down inhibited tumor cell migration and invasion.** trans-well migration and invasion assays in NEDD9 siRNA-MDA-MB-231 cells with or without 12 µM mitomycin C (MMC).
(TIF)

Figure S2 **Suppression of NEDD9 expression inhibited tumor cell migration and invasion.** Migration and invasion assays upon NEDD9 knockdown in HCC1937 cells. The migration and invasion ability is presented as fold changes in number of cells migrated to the bottom chamber. Each bar represents the mean SEM of samples measured in triplicate, and each experiment was repeated at least three times.
(TIF)

Figure S3 **NEDD9 increased the expression and secretion of MMP-9.** A, Identification of the gelatinolytic enzymes produced by NEDD9-MCF10A and control cells. The conditioned media and the cellular extracts were collected, centrifuged and the proteins were analyzed by zymography in gelatin-embedded SDS polyacrylamide gels. B, Western analysis showing the effect of NEDD9 overexpression on MMP-9. Cell lysates were prepared and subjected to western analysis using an anti-MMP-9 polyclonal antibody. β-actin was used as the loading reference.
(TIF)

Figure S4 **ChIP assays at the E-cadherin promoter.** Increased binding of HDAC1 and HDAC2 at the E-cadherin promoter in the presence of NEDD9. Primer 1 and 2 were used to amplify the E-cadherin promoter regions from −600 to −329 and −359 to −63, respectively.
(TIF)

Table S1 **Patient and tumor characteristics of 84 cases of primary invasive breast carcinoma analyzed by immunohistochemistry.**
(DOC)

Methods S1 **Quantitative RT-PCR.**
(DOC)
