# Supplementary figures and images for: NEDD9 Is a Positive Regulator of Epithelial-Mesenchymal Transition and Promotes Invasion in Aggressive Breast Cancer
Source: PLoS One. 2011 Jul 28;6(7):e22666. doi: 10.1371/journal.pone.0022666 (PMC3145662; doi:10.1371/journal.pone.0022666)

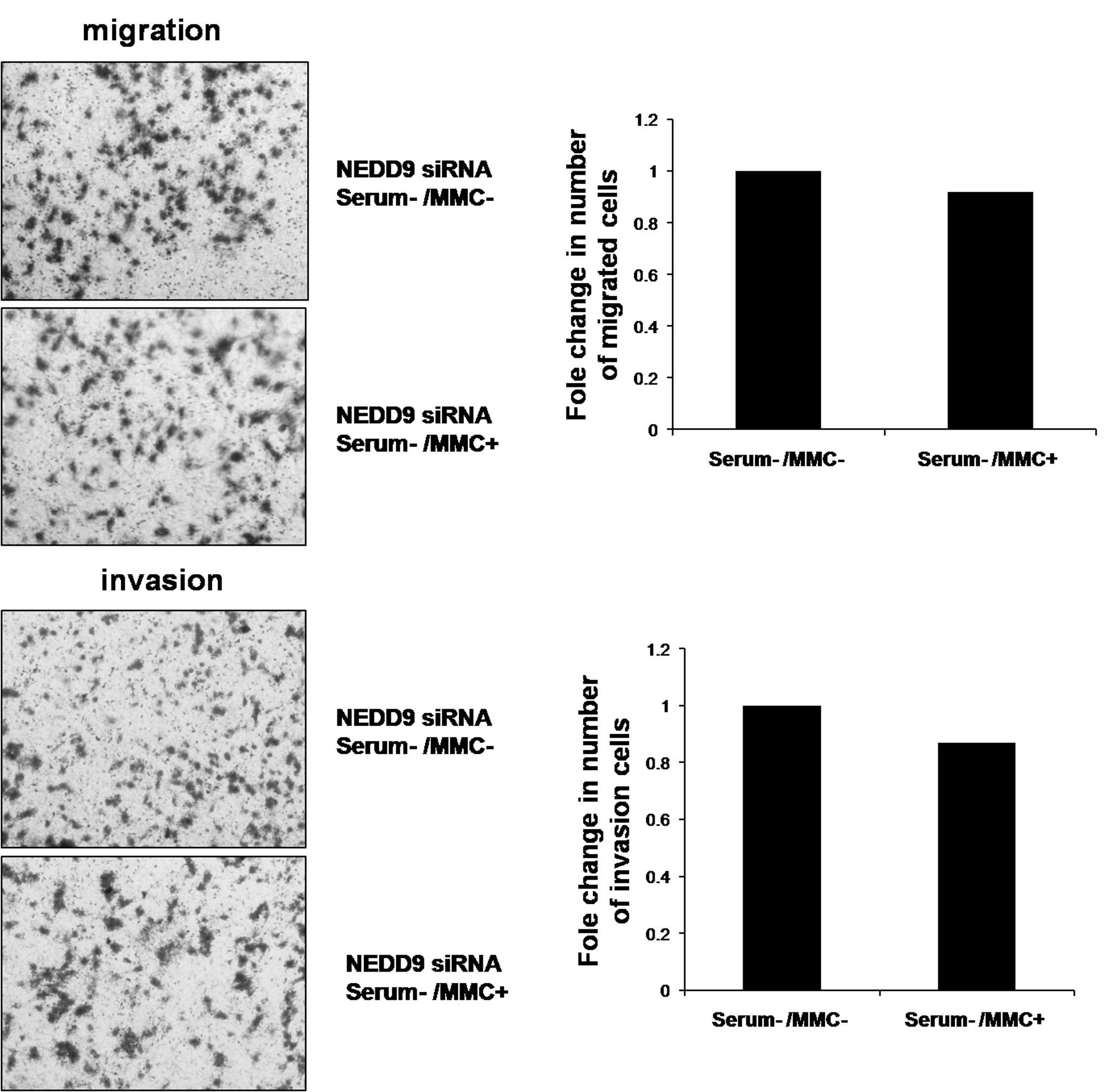

Supplement: Figure S1 — NEDD9 knock down inhibited tumor cell migration and invasion. trans-well migration and invasion assays in NEDD9 siRNA-MDA-MB-231 cells with or without 12 µM mitomycin C (MMC). (TIF) [file pone.0022666.s001.tif]

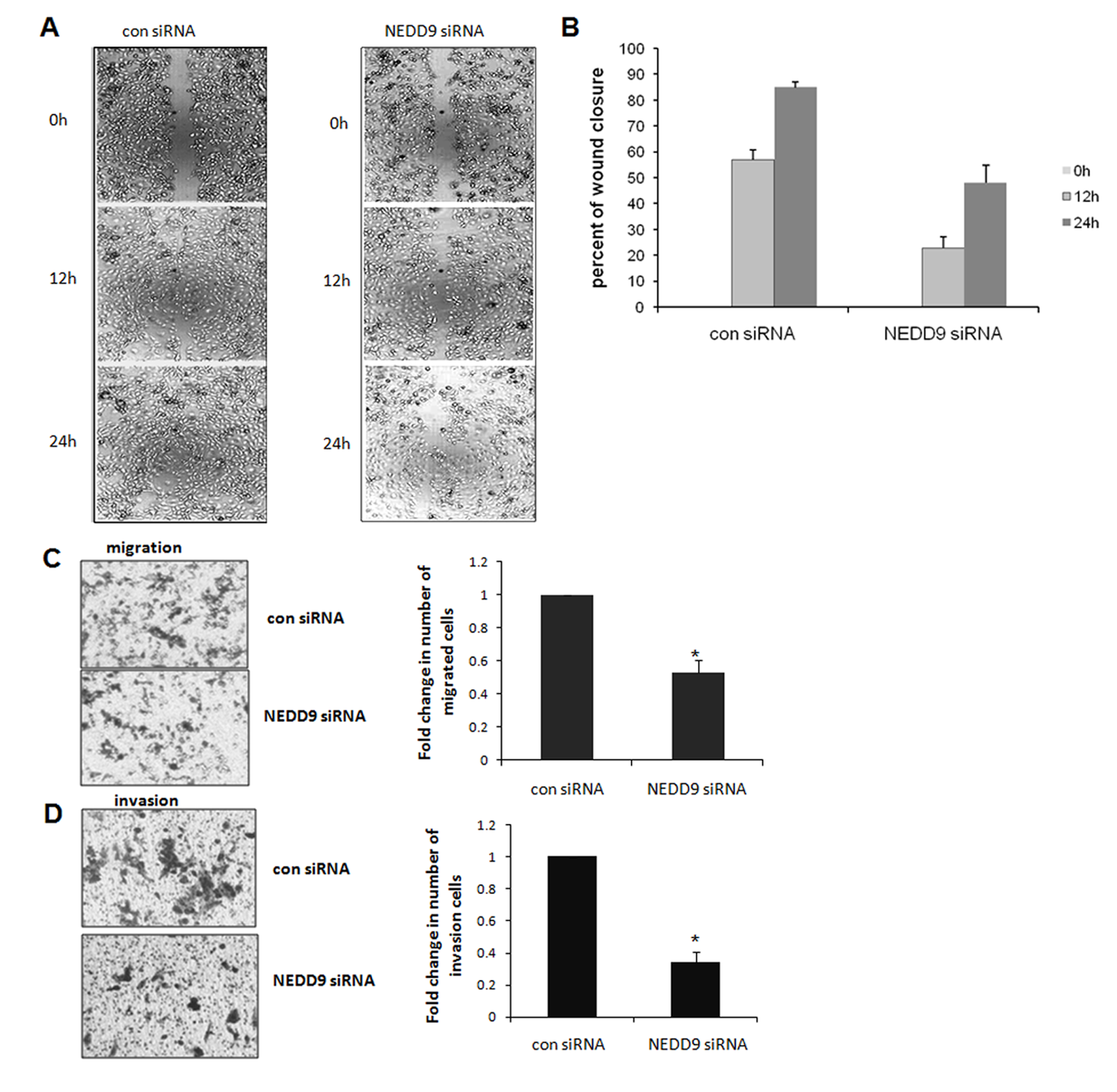

Supplement: Figure S2 — Suppression of NEDD9 expression inhibited tumor cell migration and invasion. Migration and invasion assays upon NEDD9 knockdown in HCC1937 cells. The migration and invasion ability is presented as fold changes in number of cells migrated to the bottom chamber. Each bar represents the mean SEM of samples measured in triplicate, and each experiment was repeated at least three times. (TIF) [file pone.0022666.s002.tif]

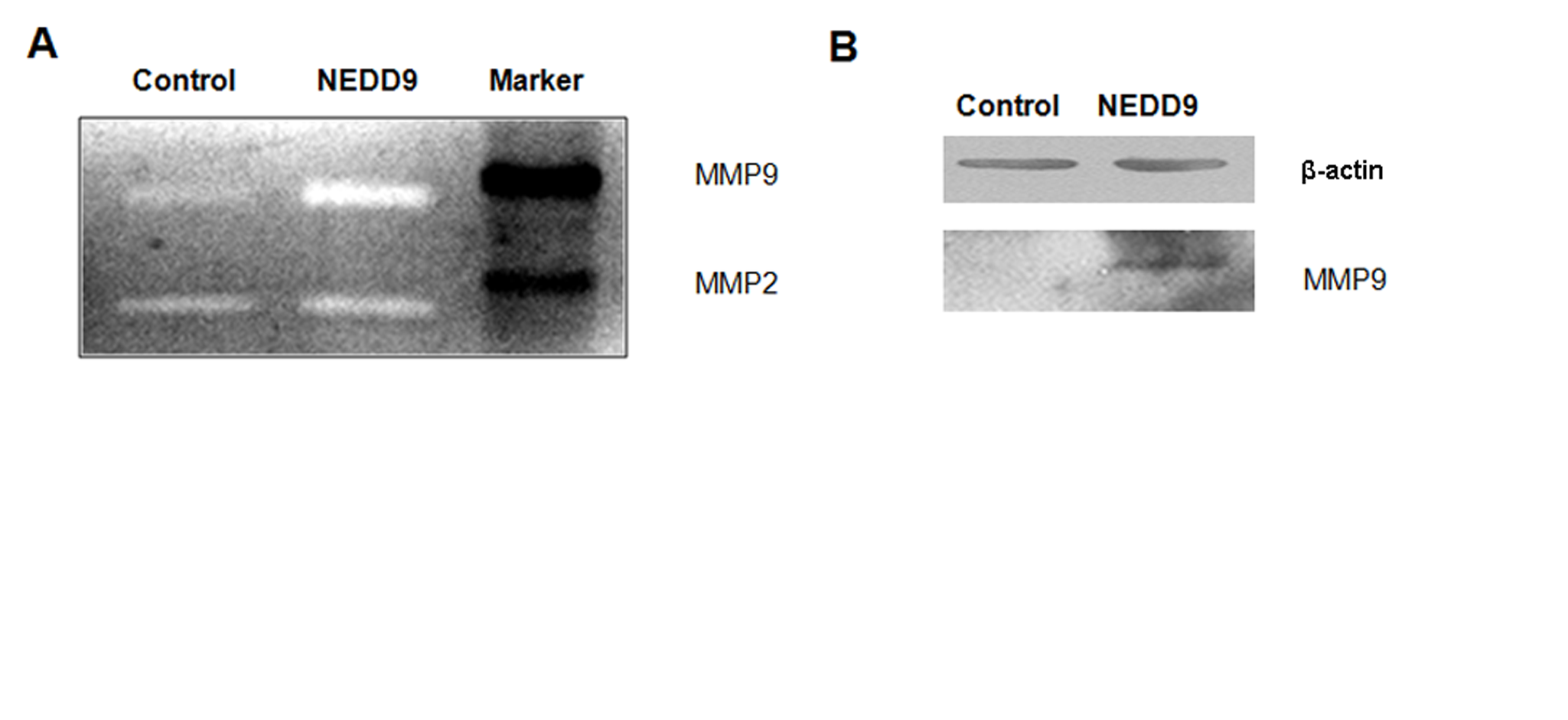

Supplement: Figure S3 — NEDD9 increased the expression and secretion of MMP-9. A, Identification of the gelatinolytic enzymes produced by NEDD9-MCF10A and control cells. The conditioned media and the cellular extracts were collected, centrifuged and the proteins were analyzed by zymography in gelatin-embedded SDS polyacrylamide gels. B, Western analysis showing the effect of NEDD9 overexpression on MMP-9. Cell lysates were prepared and subjected to western analysis using an anti-MMP-9 polyclonal antibody. β-actin was used as the loading reference. (TIF) [file pone.0022666.s003.tif]

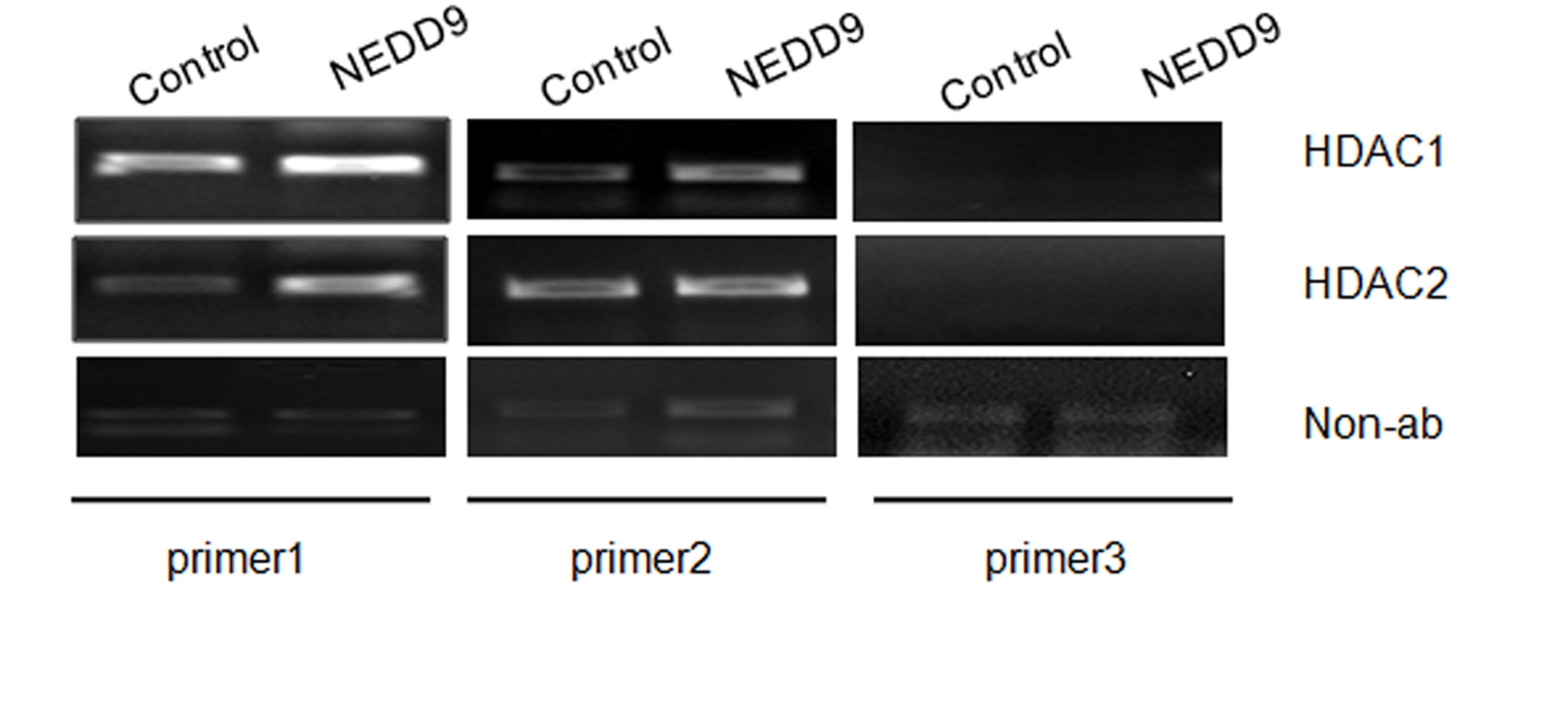

Supplement: Figure S4 — ChIP assays at the E-cadherin promoter. Increased binding of HDAC1 and HDAC2 at the E-cadherin promoter in the presence of NEDD9. Primer 1 and 2 were used to amplify the E-cadherin promoter regions from −600 to −329 and −359 to −63, respectively. (TIF) [file pone.0022666.s004.tif]
