# Supplementary material for: NEDD9 Is a Positive Regulator of Epithelial-Mesenchymal Transition and Promotes Invasion in Aggressive Breast Cancer
Source: PLoS One. 2011 Jul 28;6(7):e22666. doi: 10.1371/journal.pone.0022666 (PMC3145662; doi:10.1371/journal.pone.0022666)
Supplement: Table S1 — Patient and tumor characteristics of 84 cases of primary invasive breast carcinoma analyzed by immunohistochemistry. (DOC) [file pone.0022666.s005.doc]

**Table S1 Patient and tumor characteristics of 84 cases of primary invasive breast carcinoma analyzed by immunohistochemistry**

| **Parameter** | **Cases, n (%)** | **NEDD9^+^，n (%)** |
| --- | --- | --- |
| No. of patients | 84 | 18 |
| pathologic stage |  |  |
| Ⅰ | 5 (5.9) | 0 (0) |
| Ⅱ | 39 (46.4) | 5 (12.8) |
| Ⅲ | 40 (47.6) | 13 (32.5) |
| tumor size, cm |  |  |
| ≤2 | 41 (48.8) | 3 (7.3) |
| ＞2 | 43 (51.2) | 15 (34.9) |
| lymph node status |  |  |
| negative | 45 (53.6) | 7 (15.6) |
| positive | 39 (46.4) | 11 (28.2) |
| ER status |  |  |
| negative | 41(48.8) | 13 (31.7) |
| positive | 43(51.2) | 5 (11.6) |
| PR status |  |  |
| negative | 48 (57.1) | 15 (31.3) |
| positive | 36 (42.9) | 3 (8.3) |
| HER2/neu status |  |  |
| negative | 59 (70.2) | 12 (20.3) |
| positive | 25 (29.8) | 6 (24.0) |
