# Supplementary material for: NEDD9 Is a Positive Regulator of Epithelial-Mesenchymal Transition and Promotes Invasion in Aggressive Breast Cancer
Source: PLoS One. 2011 Jul 28;6(7):e22666. doi: 10.1371/journal.pone.0022666 (PMC3145662; doi:10.1371/journal.pone.0022666)
Supplement: Methods S1 — Quantitative RT-PCR. (DOC) [file pone.0022666.s006.doc]

**Supplementary Materials and Methods**

*Quantitative RT-PCR*

The PCR primer sequences used were as follows.

Nedd9: 5’-CTACAGGGTAAGGAGGAGTTT-3’ (sense),

5’-TGGGTCTCACATTGGTCAT-3’ (antisense);

Snail1: 5’-GCAAATACTGCAACAAGG-3’ (sense),

5’-GCACTGGTACTTCTTGACA-3’ (antisense);

Slug: 5’-AGATGCATATTCGGACCCAC-3’ (sense),

5’-CCTCATGTTTGTGCAGGAGA -3’ (antisense);

ZEB1: 5’-TGCACTGAGTGTGGAAAAGC-3’ (sense),

5’-TGGTGATGCTGAAAGAGACG-3’ (antisense);

ZEB2: 5’-CGCTTGACATCACTGAAGGA-3’ (sense),

5’-CTTGCCACACTCTGTGCATT-3’ (antisense);

Twist: 5’-GGAGTCCGCAGTCTTACGAG-3’ (sense),

5’-TCTGGAGGACCTGGTAGAGG-3’ (antisense);

β-actin: 5’-TCGTGCGTGACATTAAGGAG-3’ (sense),

5’-ATGCCAGGGTACATGGTGGT-3’ (antisense).

*Gelatin zymogram assay*

Cells were cultured in serum-free DMEM/F12 medium for 48 hr. Gelatinolytic activity of the conditioned medium was determined by gelatin zymogram assay as previously described [44]. Areas of gelatinase activity were detected as clear bands against the blue-stained gelatin background.
